# The relationship between physical activity and Internet addiction among Chinese adolescents: exploring latent profile analysis and multi-level mediating mechanisms

**DOI:** 10.3389/fpsyg.2025.1628586

**Published:** 2025-07-09

**Authors:** Jiamin Zhu, Yutong Zhai, Xiaotong Yuan, Zhiyong Zhang, Xiaoping Meng

**Affiliations:** ^1^Graduate School, Shandong Sport University, Jinan, China; ^2^College of Economics and Management, Nanjing University of Aeronautics and Astronautics, Nanjing, China; ^3^Institute of Physical Education and Social Sciences, Shandong Sports University, Jinan, China; ^4^School of Sport Management, Shandong Sport University, Jinan, China

**Keywords:** Internet addiction, physical activity, family cohesion, prosocial behavior, latent profile analysis

## Abstract

In recent years, adolescent Internet addiction has emerged as a pressing concern, undermining young people’s psychological well-being and social functioning. Although regular physical activity is known to bolster adolescents’ mental health, its direct and indirect protective effects against Internet addiction—and the psychosocial processes involved—remain insufficiently understood, particularly in terms of how these effects differ across subgroups identified via latent profile analysis. Given this, the present study, employing structural equation modeling (SEM), delineated the factor structures and predictive relationships among physical activity, family cohesion, prosocial behavior, and Internet addiction. Moreover, to investigate how these pathways vary across different user groups, we conducted latent profile analysis (LPA) to identify distinct Internet-use typologies and tested their Mediation effects. The measurement model supported adequate construct validity and internal consistency across all latent variables, ensuring the reliability of subsequent structural analyses. SEM results demonstrated that physical activity exerted a significant negative direct effect on Internet addiction (β = −0.227, *p* < 0.001), and indirect effects via family cohesion (−0.065, 15.7% of total effect) and prosocial behavior (−0.083, 19.9% of total effect), as well as a chained pathway from family cohesion to prosocial behavior (−0.043, 10.1% of total effect). LPA supported a three-class solution—Functional Use (31.2%), At-Risk Use (49.5%), and Addicted Use (19.3%)—and multi-group SEM indicated that the magnitude of both indirect pathways varied across these typologies, with the strongest mediation observed in the At-Risk group. These findings suggest that physical activity is associated with reduced adolescent Internet addiction both directly and indirectly through socio-psychological mechanisms, and that intervention efforts should be tailored to specific user typologies to maximize preventive and remedial impact. These findings suggest that school-based physical activity interventions may help mitigate Internet addiction among Chinese adolescents.

## 1 Introduction

Adolescence is a critical developmental stage, yet contemporary youth engage with the Internet at unprecedented rates. Recent national statistics reveal that adolescents constitute 26% of all Internet users in China, and Internet penetration among secondary-school students has reached 91.8% (Liu et al., 2023; Zhou et al., 2025). Though online connectivity offers obvious educational and social advantages, epidemiological studies indicate that 6–14% of teenagers meet clinical-risk criteria for Internet addiction—a compulsive behavior marked by inattention, emotional instability, reduced self-control, and social withdrawal, all of which compromise mental health and daily functioning (Ying et al., 2024).

Previous studies have consistently shown that harsh or inconsistent parenting, limited parental monitoring, adverse childhood experiences, internalizing symptoms, and deficits in self-regulation increase adolescents’ susceptibility to Internet addiction (Davies et al., 2019; Skoranski et al., 2022). Most prevention efforts still focus on individual-level skill development—enhancing digital self-efficacy, strengthening self-control, and refining emotion-regulation capacities—and are generally implemented only after early warning signs of excessive use have appeared (Lincoln et al., 2022; Irmayanti and Chusniyah, 2024). However, the empirical foundation of these programs primarily relies on variable-centered analyses that report only average effects, thereby obscuring latent typological differences in adolescents’ online behavior (Gini et al., 2019; Remondi et al., 2024). Unlike variable-centered methods, LPA identifies subgroups within heterogeneous populations, offering more tailored insight into distinct behavioral patterns. Consequently, focusing on average trends neglects earlier-intervening interpersonal and contextual factors that require closer scrutiny.

Positive mental health outcomes in young people have been linked to family cohesion, which is characterized by emotional closeness, supportive communication, and consistent behavioral norms shared among family members (Qi et al., 2023). When physical activity is incorporated into family routines (e.g., parent–child sports, weekend excursions), it provides opportunities for mutual encouragement and shared goals, thereby strengthening these cohesive bonds and promoting balanced media habits as well as effective screen-time monitoring (Bates et al., 2020). Additionally, prosocial behavior—which is characterized by voluntary acts of cooperation, empathy, and helping—is encouraged by physical activity (Wan et al., 2021). Team-based physical activity requires collaboration and fair-play norms, fostering empathy and a sense of belonging that reduce the appeal of virtual gratification (Cheng K. H. et al., 2024). Together, enhanced family cohesion and increased prosocial engagement create an interlocking protective network that redirects adolescents from compulsive online behavior toward healthier, offline sources of competence and connection.

The current study examines whether physical activity is indirectly linked to the severity of Internet addiction through prosocial behavior and family cohesion, guided by ecological systems and self-determination perspectives. We use latent profile analysis (LPA) to find unique patterns of teenage Internet use in order to capture behavioral heterogeneity that variable-centered approaches miss. This research attempts to improve knowledge of the psychological pathways connecting physical activity, family dynamics, and adolescent Internet addiction by combining multilevel theory with a person-centered analytical approach. This will provide an empirical basis for prevention initiatives that are based on activities and families.

## 2 Theoretical foundations and research hypotheses

### 2.1 The predictive role of physical activity on internet addiction

Internet addiction is characterized as an impulse control disorder resulting from prolonged and excessive internet use, marked by compulsive engagement with the internet and impairments in real-life functioning (Weinstein and Lejoyeux, 2010; Cerniglia et al., 2017). Physical activity is increasingly acknowledged as a crucial factor in promoting both physical and mental health (Faulkner et al., 2021). It plays a critical role in enhancing emotional regulation, self-control, and overall psychological resilience, all of which are essential for managing internet addiction. According to Self-Determination Theory (SDT), physical activity fulfills basic psychological needs, particularly those related to competence, autonomy, and relatedness, which are fundamental to well-being and personal growth (Górnik-Durose et al., 2018). When adolescents engage in physical activity, they experience a sense of mastery and achievement, which enhances their confidence in managing daily challenges and stressors. This sense of competence can directly counteract the desire for maladaptive coping strategies, such as excessive internet use (Lara et al., 2021).

Additionally, physical activity has been demonstrated to improve self-regulation and emotional stability. Self-regulation refers to the ability to control one’s emotions, behaviors, and impulses in response to external demands, and it plays a critical role in preventing addiction. Adolescents who engage in regular physical activity are more likely to develop effective coping strategies and emotional regulation skills, thereby reducing their reliance on the internet for emotional relief. This concept aligns with the Compensatory Internet Use Theory (Yin and Shen, 2023), which posits that individuals often turn to the internet as a coping mechanism to satisfy unmet psychological needs, such as emotional support or self-validation (Snodgrass et al., 2018). When adolescents engage in physical activity, they are more likely to fulfill these needs through healthier means, thus reducing their tendency to use the internet as a form of escape. Furthermore, empirical studies support the role of physical activity in mitigating addictive behaviors (Millstein, 2020). Adolescents who engage in regular physical activity report higher life satisfaction, improved emotional well-being, and better social adaptation—all of which are essential in preventing internet addiction (Liu et al., 2021). Research has shown that physical activity not only enhances cognitive regulation and life satisfaction but also strengthens interpersonal relationships by fostering social engagement and positive social interactions outside the virtual world. These outcomes help reduce the risk of internet addiction by promoting healthier coping mechanisms and emotional resilience (Du and Zhang, 2022; Qiu et al., 2023). Based on these findings, we hypothesize that physical activity is negatively associated with the tendency toward internet addiction among middle school students.

### 2.2 The mediating role of family cohesion

According to ecological systems theory, the family, as the primary context for individual socialization, plays a pivotal role in shaping adolescents’ behaviors through its emotional environment (Katz-Wise et al., 2022). Family cohesion, which includes emotional bonds, communication quality, and the level of support shared among family members, is recognized as a critical protective factor against various adolescent problem behaviors, such as aggression, depression, and addiction (Deković, 1999; Monahan et al., 2014). Previous research suggests that family cohesion fosters emotional stability and security while also serving as a buffer against negative psychological outcomes (Rahal and Fosco, 2025). Recent studies have highlighted the role of physical activity in enhancing adolescents’ psychological resilience and strengthening family cohesion. Physical activity has been shown to enhance emotional bonding by promoting positive interactions among family members. When family members engage in physical activities together, adolescents are more likely to perceive increased cohesion and support within the family, which in turn reduces their tendency to seek emotional compensation through virtual networks and internet use (Li N. et al., 2024; Li Z. et al., 2024). This implies that engaging in physical activity could help decrease the likelihood of internet addiction by fostering family cohesion (Kuss and Griffiths, 2011). Prior research suggests gender moderates the relationship between family dynamics and online behavior, indicating the need to consider individual differences in future investigations.

Family cohesion seems to mediate the link between physical activity and internet addiction given its important part in offering emotional support and stability. By fostering stronger family connections, physical activity may reduce the risk of adolescents turning to the internet as a coping mechanism.

### 2.3 The mediating role of prosocial behavior

Prosocial behavior refers to voluntary actions intended to benefit others, such as helping, sharing, and cooperating, and is a critical indicator of an individual’s social adaptation (Baldassarri and Abascal, 2020). Adolescents who engage in prosocial behaviors are more likely to form positive social relationships, which contribute to their emotional and social well-being. Recent studies have shown that physical activity, by providing opportunities for social interaction and teamwork, promotes the development of prosocial behaviors (Liang et al., 2025). Engaging in physical activities such as team sports or group physical activity enables adolescents to collaborate, communicate, and build supportive relationships, which enhances their ability to exhibit prosocial behaviors both within and outside the activity context (Wentzel and Watkins, 2002). According to Self-Determination Theory (SDT), shared physical activity helps adolescents satisfy their need for relatedness. Fulfilling this need can promote empathy, cooperation, and prosocial tendencies. From the perspective of social learning theory (Rumjaun and Narod, 2025), adolescents acquire behaviors through observation and interaction with others in their social environment. Physical activity, particularly in a group or team setting, provides an ideal context for acquiring prosocial behaviors. Adolescents observe and model the cooperative, helping behaviors of their peers and coaches, reinforcing the value of these actions through positive social feedback and reinforcement. These learned behaviors become integrated into their social repertoire, leading to increased engagement in prosocial actions both during and after physical activity sessions (Li and Shao, 2022).

Furthermore, adolescents who engage in prosocial behavior are more likely to seek recognition and validation through real-world interactions (El Mallah, 2020), thereby reducing their reliance on virtual environments for social affirmation. This shift toward real-world connections may reduce the tendency to use the internet as a coping mechanism for unmet emotional or social needs (Kardefelt-Winther, 2014).

Given these theoretical insights, it is reasonable to think that prosocial behavior mediates the connection between physical activity and internet addiction. Physical activity helps teenagers to create prosocial behaviors that improve social adaptation and emotional resilience, so lowering the probability of seeking the internet for social or emotional compensation.

### 2.4 The chained mediating role of family cohesion and prosocial behavior

From a socio-ecological and motivational perspective, family cohesion and prosocial behavior should be considered interconnected components within a progressive psychological pathway rather than independent constructs. Family cohesion, defined by strong emotional bonds, effective communication, and mutual support among family members, creates a secure environment that fosters adolescents’ emotional and social development (Xiang et al., 2022). According to Family Systems Theory, family cohesion facilitates adaptive functioning by promoting emotional support and clear communication, crucial elements in nurturing adolescents’ prosocial tendencies (Gao et al., 2024). Additionally, Self-Determination Theory posits that cohesive family environments satisfy adolescents’ fundamental psychological needs for relatedness, competence, and autonomy, fostering intrinsic motivation to engage in prosocial behaviors (Rivas and Albertos, 2023). Adolescents from cohesive families are more likely to develop these positive traits, enhancing their social adaptation and contributing to emotional stability (Abdul Kadir, 2024; Gao et al., 2024).

Conversely, adolescents who exhibit higher levels of prosocial behavior are less likely to engage in maladaptive coping mechanisms, such as excessive internet use, as they tend to seek real-world validation and social connections (Memmott-Elison et al., 2022). This shift away from virtual interactions is particularly crucial in reducing reliance on the internet for emotional compensation, which can otherwise contribute to internet addiction (Hu et al., 2022). Social Compensation Theory suggests that individuals lacking sufficient offline social support are more likely to seek emotional fulfillment online (van Ingen and Wright, 2016), thereby linking prosocial behaviors to decreased internet addiction risk.

Therefore, physical activity may play a crucial role in reducing adolescents’ risk of internet addiction through a chained mediating pathway: increased family cohesion enhances prosocial behavior, which subsequently decreases the likelihood of excessive internet use. By engaging in physical activities that promote social interaction and cooperation, adolescents experience improvements in their family dynamics and social behaviors, further reducing their reliance on virtual networks for emotional fulfillment. This pathway underscores how physical activity can indirectly influence adolescents’ internet addiction tendencies by strengthening family cohesion and promoting prosocial behaviors. Based on this reasoning, we propose that physical activity indirectly influences internet addiction through the chained mediating pathway of family cohesion and prosocial behavior.

### 2.5 The role of latent profile analysis in understanding internet addiction

Adolescents’ internet use behaviors are not uniform; instead, they exhibit considerable heterogeneity across dimensions such as addiction tendency, behavioral control, and psychological dependence. Previous studies suggest that adolescent internet behaviors vary significantly (Bickham, 2021b), with some individuals exhibiting higher addiction tendencies while others demonstrate more controlled and adaptive use (Deleuze et al., 2015). Latent Profile Analysis (LPA), a sophisticated statistical technique, enables researchers to identify latent subgroups within a population based on multiple dimensions, including factors such as physical activity, family cohesion, and prosocial behavior (Shek, 1997; Young et al., 2007). This approach provides a deeper understanding of the complexities of adolescent internet use, revealing distinct profiles that traditional analyses may overlook (Chen and Fan, 2024). For instance, adolescents with higher levels of internet addiction may rely more on the protective effects of family cohesion, with strong emotional support from family members acting as a buffer against the negative consequences of excessive internet use. In contrast, adolescents who exhibit less problematic internet use, or those categorized as functional users, may be more influenced by the prosocial behavior pathway (Liang et al., 2025), in which family cohesion fosters cooperative and empathetic behaviors that reduce the risk of developing internet addiction.

By applying latent profile analysis, we can uncover distinct behavioral subgroups, revealing how physical activity and family dynamics relate differently to adolescents’ patterns of internet use. Given differing levels of self-regulation and emotional coping resources, the strength of these mediating pathways may vary by internet use severity. Accordingly, we hypothesize that these latent profiles will differ notably in their levels of physical activity, family cohesion, and prosocial behavior, and that the pathways linking physical activity to internet addiction will vary across profiles.

**Figure 1:**
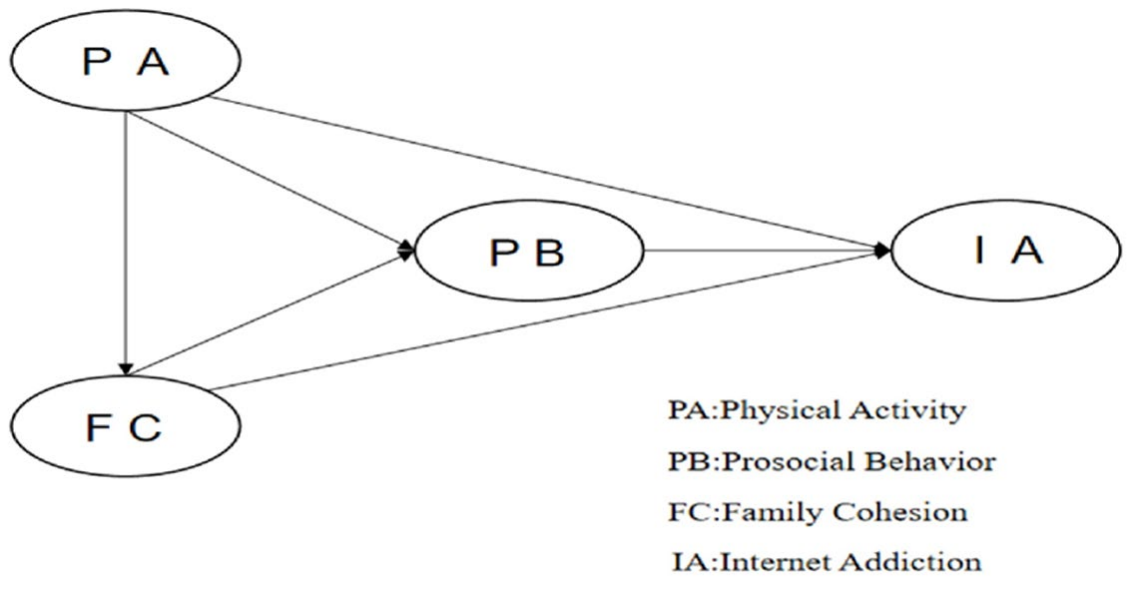
Hypothesized model.

While previous research has examined the effects of physical activity, family cohesion, and prosocial behavior on adolescent well-being independently, there are few studies that have explored their combined impact on problematic internet use. This study aims to investigate how physical activity predicts tendencies toward internet addiction in adolescents, with family cohesion and prosocial behavior serving as potential mediators. Utilizing ecological systems theory and self-determination theory, we propose the model illustrated in Figure 1, which outlines these hypothesized relationships.

We utilize latent profile analysis (LPA) to categorize teenagers into distinct groups based on their internet usage, allowing us to examine how the impacts on these groups vary. By combining LPA with ecological systems theory and self-determination theory, this study shows the different ways that physical activity, family closeness, and helping behavior can lead to internet addiction in teenagers. The following hypotheses are presented:

*H1*. Physical activity is negatively associated with the tendency toward internet addiction.

*H2*. Family cohesion mediates the relationship between physical activity and the tendency toward internet addiction.

*H3*. Prosocial behavior mediates the relationship between physical activity and the tendency toward internet addiction.

*H4*. Physical activity negatively predicts the tendency toward internet addiction through the mediated pathway of family cohesion and prosocial behavior.

*H5*. There are multiple latent types of adolescent internet addiction behaviors, with significant differences in levels of physical activity, family cohesion, and prosocial behavior across these types.

*H6*. The mechanism through which physical activity influences internet addiction via the family cohesion → prosocial behavior pathway varies across different types of internet use.

## 3 Research methodology

### 3.1 Participants

Between October 12 and December 30, 2024, we recruited students from three public middle schools in Shandong Province using a stratified random sampling approach. In total, 750 questionnaires were administered, yielding 690 valid responses (334 males, 48.4%; 356 females, 51.6%). The final sample included 690 adolescents from three public secondary schools (Mage = 13.51, SD = 0.93, range = 12–15). Socioeconomic background was described based on parental education and residential location. Regarding education, 21.7% of fathers and 26.1% of mothers had a college degree or above. In terms of residence, 68.4% of participants lived in urban areas, while 31.6% resided in rural or township regions. The study focuses on adolescent internet addiction behaviors, referencing national analysis estimates of prevalence ranging from 6.92 to 8% (Han et al., 2021). The median value of 8% was chosen as the estimated prevalence (P) for sample size calculation. The researchers calculated the sample size using Cochran’s formula for proportions: N = Z^2^ × P(1 − P) / *δ*^2^, where N is the required sample size, Z is the z-score corresponding to the desired confidence level (1.96 for α = 0.05), P is the estimated prevalence (0.08), and δ is the permissible margin of error (0.04) (Cochran, 1977). Substituting the values results in 1.96^2^ × 0.08 × (1–0.08) / 0.04^2^ ≈ 177. We adjusted the final required sample size to 197 participants, taking into account a 10% invalid response rate. Schumacker and Lomax review literature showing that many structural equation modeling studies use sample sizes ranging from 250 to 500, which is considered an appropriate sample size for such analyses (Lomax, 2004). In LPA analysis, having between 200 and 500 participants is good for looking at variables, but having fewer than 200 can cause problems with the results and make it hard to see smaller individual traits (Meyer and Morin, 2016). This sample size is sufficient to ensure the statistical power required for our analyses.

This study was conducted following the principles outlined in the Declaration of Helsinki. Before data collection commenced, we obtained ethical approval and administrative consent from the Ethics Committee of the Institute of Sport Social Sciences at Shandong Sport University (Ethical Approval No. 2024023). Before data collection, all participants (and their parents, in the case of minors) provided written informed consent after being briefed on the study’s objectives, procedures, potential risks, and confidentiality safeguards. Participation was entirely voluntary, and participants were free to withdraw at any point without consequence. Teachers also informed parents about the survey and facilitated their consent process. All data were anonymized and used exclusively for research purposes.

### 3.2 Measurement tools

#### 3.2.1 Physical activity rating scale

A Physical activity was assessed with the Physical Activity Rating Scale (PARS-3), a Chinese adaptation by Liang Deqing of Hashimoto Kimio’s original instrument (Liang and Liu, 1994). The PARS-3 evaluates physical activity intensity, frequency, and duration, yielding a composite activity score calculated as Activity volume = Intensity × (Duration – 1) × Frequency. Higher scores indicate greater overall engagement in physical activity. In our sample, the PARS-3 demonstrated excellent internal consistency (Cronbach’s α = 0.912), supporting the scale’s reliability and construct validity. Previous studies have similarly demonstrated that the PARS-3 maintains strong reliability and validity among adolescent populations (Yang G. et al., 2021).

#### 3.2.2 Internet addiction test

Students’ internet addiction was measured with Young’s Internet Addiction Test (IAT) (Young, 2009), a 20-item questionnaire using a 5-point Likert scale (1 = rarely to 5 = always), yielding total scores from 20 to 100, with higher scores indicating stronger addictive tendencies. In this study, the IAT showed excellent internal consistency (Cronbach’s α = 0.910) and demonstrated good construct validity. Based on Chinese normative criteria, scores of 20–49 reflect normal internet use, while scores of 50 or above indicate internet addiction. Previous research has likewise suggested that the IAT possesses strong reliability and validity across diverse adolescent samples (Hawi, 2013).

#### 3.2.3 Family cohesion scale

Family cohesion was assessed with the cohesion subscale of the Chinese version of the Family Adaptability and Cohesion Evaluation Scale (FACES II-CV), as translated and adapted by Fei Li peng et al. (Phillips et al., 1998). This subscale comprises 30 items rated on a 5-point Likert scale, where higher scores denote stronger emotional bonds among family members. In the current sample, the cohesion subscale demonstrated excellent internal consistency (Cronbach’s α = 0.923) and supported strong construct validity, Previous studies have likewise suggested that this scale exhibits good reliability and validity in measuring family cohesion (Cheng J. et al., 2024).

#### 3.2.4 I adolescents’ prosocial behavior measure

Adolescents’ prosocial behavior was assessed using the Prosocial Tendency Measure (PTM), revised by Kou et al. (2007). The scale comprises 26 items rated on a 5-point Likert scale (1 = strongly disagree to 5 = strongly agree), with higher scores indicating a stronger prosocial tendency. The PTM has demonstrated strong reliability and validity in adolescent samples (Yang Y. et al., 2021), and in the present study it achieved excellent internal consistency (Cronbach’s α = 0.974). Previous research has likewise suggested the PTM’s robust psychometric properties in youth populations.

### 3.3 Data analysis methods

Data management and analysis were conducted using SPSS 26.0, AMOS 27.0, and Mplus 8.3. Specifically, SPSS was used for descriptive statistics, correlations, and regression analyses; AMOS was used for structural equation modeling (SEM) and multi-group analysis; and Mplus was used for latent profile analysis (LPA). To begin, we tested for common method bias via Harman’s single-factor approach. We then computed descriptive statistics, Pearson correlations, and regression analyses to explore bivariate relationships. Controlling for gender and internet use behaviors, we evaluated model fit through structural equation modeling (SEM) and examined mediation effects. Model fit was deemed acceptable according to conventional thresholds: χ^2^/df < 3, RMSEA ≤ 0.08, SRMR ≤ 0.10, and GFI, CFI, TLI, AGFI > 0.90 (Kline, 2023). Next, we conducted latent profile analysis (LPA) in Mplus using IAT items as indicators, fitting models with one to five classes. Unlike traditional variable-centered approaches that focus on average effects, LPA uncovers hidden subgroups with distinct patterns of internet-use behavior, offering a richer view of adolescent addiction typologies. Model selection relied on AIC, BIC, aBIC, entropy, the Lo–Mendell–Rubin test (LMRT), and the Bootstrap Likelihood Ratio Test (BLRT) to ensure optimal fit. We then treated the resulting classes as groups in a multi-group structural equation modeling (SEM) analysis in AMOS to compare mediation pathways—from physical activity through family cohesion and prosocial behavior to internet addiction—across profiles. By incorporating multiple mediators and using the bias-corrected percentile bootstrap method (10,000 resamples) to generate 95% confidence intervals, this approach captures the complex indirect effects more accurately than traditional single-path models.

## 4 Research results

### 4.1 Common method bias test

Common method bias was assessed using Harman’s single-factor test via an unrotated exploratory factor analysis on all study items. Eight factors with eigenvalues greater than one emerged, and the first factor explained 27.61% of the total variance—well below the 40% threshold (Kock et al., 2021). These results indicate that common method bias is minimal and that the measures retain adequate discriminant validity.

### 4.2 Descriptive statistics and correlation analysis of variables

**Figure 2:**
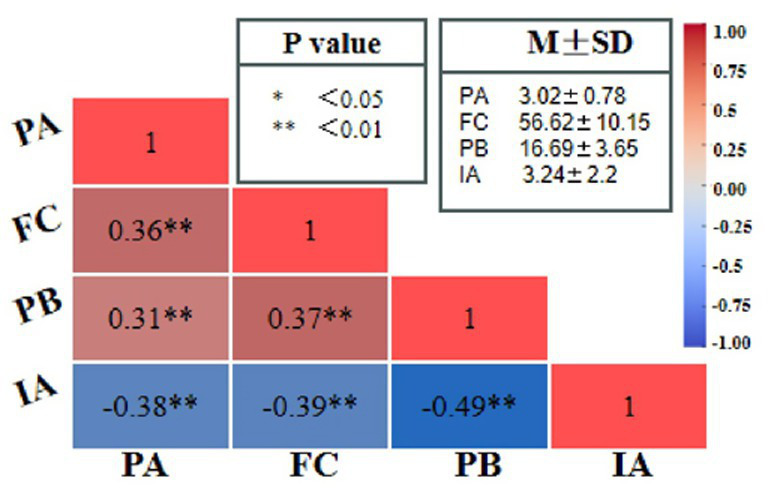
Correlation matrix among variables.

We calculated basic statistics (like averages and standard deviations) and Pearson correlation coefficients for the four main factors—physical activity (PARS-3), family cohesion (FACES II-CV), prosocial behavior (PTM), and internet addiction (IAT)—as shown in Figure 2. Physical activity was positively associated with family cohesion (*r* = 0.36, *p* < 0.01) and prosocial behavior (*r* = 0.31, *p* < 0.01) and negatively related to internet addiction (*r* = −0.39, *p* < 0.01). Family cohesion showed a positive correlation with prosocial behavior (*r* = 0.37, *p* < 0.01) and a negative correlation with internet addiction (*r* = −0.38, *p* < 0.01). Finally, prosocial behavior and internet addiction were moderately inversely correlated (*r* = −0.49, *p* < 0.01). These patterns suggest that greater physical activity and stronger family bonds align with higher prosocial tendencies and lower levels of internet addiction.

### 4.3 Analysis of the relationship between physical activity, family cohesion, prosocial behavior, and internet addiction

After controlling for gender and grade, stepwise regression showed that physical activity alone significantly predicted lower internet addiction (β = −0.39, *t* = −11.05, *p* < 0.001), explaining 15.1% of the variance. Adding family cohesion increased explained variance by 6.5–21.6%, with both physical activity (β = −0.29, *t* = −8.01, *p* < 0.001) and family cohesion (β = −0.27, *t* = −7.57, *p* < 0.001) remaining significant. When prosocial behavior was included, all three predictors stayed significant—physical activity (β = −0.22, *t* = −6.29), family cohesion (β = −0.17, *t* = −4.75), and prosocial behavior (β = −0.36, *t* = −10.36; all *p* < 0.001)—and the model’s explanatory power rose by 11.0 to 32.6%. These results indicate that physical activity, family cohesion, and prosocial behavior each make a unique contribution to reducing adolescents’ risk of internet addiction (Table 1).

**Table 1:** The regression analysis results of physical activity, family cohesion, and prosocial behavior on Internet addiction.

Dependent variable	Model 1		Model 2		Model 3	
	β	*t*	β	*t*	β	*t*
Physical activity	−0.39	−11.05**	−0.29	−8.01**	−0.22	−6.29**
Family cohesion			−0.27	−7.57**	−0.17	−4.75**
Prosocial behavior					−0.36	−10.36**
*R* ^2^	0.151		0.216		0.322	
△*R*^2^	0.149		0.214		0.319	
*F*	122.04**		94.71**		108.65**	

*p* < 0.01 (two-tailed), statistically significant.

### 4.4 Model and mediation effect testing

**Figure 3:**
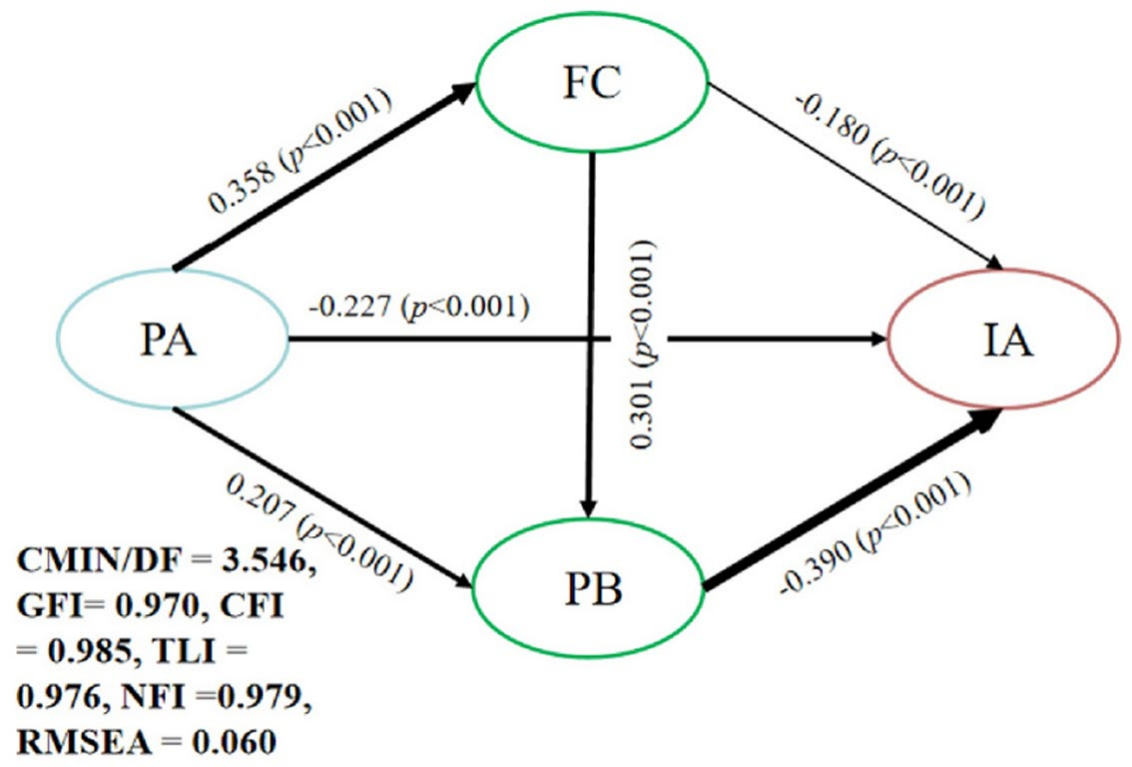
SEM test model.

To examine how physical activity influences adolescent internet addiction via family cohesion and prosocial behavior, we specified a structural equation model in AMOS 27.0 using maximum-likelihood estimation. The structural equation model demonstrated an acceptable fit to the data, with χ^2^/df = 3.546, GFI = 0.970, CFI = 0.985, TLI = 0.976, NFI = 0.979, and RMSEA = 0.060, all of which meet or exceed conventional thresholds (Kline, 2023). Physical activity was entered as the independent variable, family cohesion and prosocial behavior as mediators, and internet addiction as the outcome, with gender, grade, and only-child status included as covariates. The direct path from physical activity to internet addiction was significant and negative (β = −0.227, *p* < 0.001), supporting H1. Bootstrapped mediation analysis (10,000 resamples, 95% CI) revealed that physical activity significantly enhanced family cohesion (β = 0.358, *p* < 0.001), which in turn reduced internet addiction (β = −0.180, *p* < 0.001), yielding an indirect effect of −0.065 (15.7% of the total effect) in line with H2. Likewise, physical activity increased prosocial behavior (β = 0.207, *p* < 0.001), and prosocial behavior significantly predicted lower internet addiction (β = −0.390, *p* < 0.001), producing an indirect effect of −0.083 (19.9% of the total effect) in support of H3. In addition, family cohesion positively predicted prosocial behavior (β = 0.301, *p* < 0.001), and the chained indirect effect from physical activity through family cohesion and then prosocial behavior to internet addiction was −0.043 (10.1% of the total effect), which is consistent with the proposed Hypothesis H4. All bootstrap confidence intervals excluded zero, and effect sizes indicate that prosocial behavior serves as the strongest mediator. Path coefficients and fit indices are detailed in Figure 3 and Table 2.

**Table 2:** Bootstrap mediation analysis.

Model effect	Effect value	Effect percentage	Boot SE	Bootstrap 95%CI	
				Lower upper	
PA → IA (direct effect)	−0.227	54.6%	0.043	−0.314	−0.142
PA → FC → IA	−0.065	15.6%	0.16	−0.100	−0.035
PA → PB → IA	−0.082	19.7%	0.20	−0.128	−0.049
PA → FC → PB → IA	−0.042	10.1%	0.009	−0.063	−0.028
Indirect effect	−0.189	45.4%	–	–	–
Total effect	−0.416	100	0.041	−0.495	−0.335

### 4.5 Latent profile analysis of internet addiction groups

We used Mplus to perform latent profile analysis (LPA) on all 20 items of the Internet Addiction Test (IAT) to uncover distinct patterns of adolescents’ online behaviors. Competing models with one through five classes were evaluated (Table 3), and the three-class solution emerged as optimal, showing the lowest AIC, BIC, and aBIC values along with an entropy of 0.925, which indicates excellent classification precision. Both the Lo–Mendell–Rubin adjusted likelihood-ratio test and the bootstrap likelihood-ratio test were significant (*p* < 0.05), suggesting that three classes provided a significantly better fit than two. Each class comprised at least 10% of the sample, and the item-level probability curves were clearly distinct across profiles. Accordingly, we retained three latent groups: a Functional-Use group with uniformly low symptom scores reflecting typical internet use; an At-Risk group with moderate scores suggesting emerging problems; and an Addicted-Use group displaying high scores across all items and clear addiction symptoms. To facilitate interpretation, we calculated the average total IAT scores for each latent class. The Functional Use group (Class 1) had a mean score of 18.3, indicating consistently low symptom levels across all items and reflecting typical internet usage patterns. The At-Risk Use group (Class 2) averaged 38.7, characterized by intermediate levels of endorsement on items such as preoccupation and mood-related disruption. The Addicted Use group (Class 3) had the highest mean score of 63.2, with item-level responses generally exceeding 3.5, suggestive of frequent compulsive use and broader psychosocial interference. These quantitative profiles provide clear behavioral anchors to distinguish the three latent classes identified through LPA. These results demonstrate considerable heterogeneity in adolescent internet addiction, underscoring the value of profile-based approaches.

**Table 3:** Fitting index and group size of latent profile analysis models.

Indices	Unconditional model				
	1-Profile	2-Profile	3-Profile^a^	4-Profile	5-Profile
Fit statistics					
AIC	24761.441	21932.022	**21181.370**	20737.617	20509.381
BIC	24942.909	22208.760	**21553.379**	21204.896	21071.931
aBIC	24815.902	22015.076	**21293.016**	20877.855	20678.211
Entropy	1	0.921	**0.925**	0.874	0.877
BLRT	–	0.0000	**0.0000**	0.0000	0.0000
LMR	–	0.0000	**0.0000**	0.0052	0.0411
Group size (%)	–	–	**–**	–	–
C1	(690)100.0	309 (44.7%)	**310 (44.9%)**	195 (28.2%)	182 (26.3%)
C2	–	381 (55.3%)	**178 (25.7%)**	173 (25.1%)	199 (28.8%)
C3	–	–	**202 (29.4%)**	214 (31.1%)	160 (23.1%)
C4	–	–	**–**	108 (15.6%)	107 (15.5%)
C5	–	–	**–**	–	42 (6.3%)

AIC, Akaike Information Criterion; BIC, Bayesian Information Criterion; aBIC, adjusted Bayesian Information Criterion; LMRT, Lo–Mendell–Rubin adjusted likelihood-ratio test; BLRT, Bootstrap likelihood-ratio test.

a The best-fit solution is indicated in bold.

**Figure 4:**
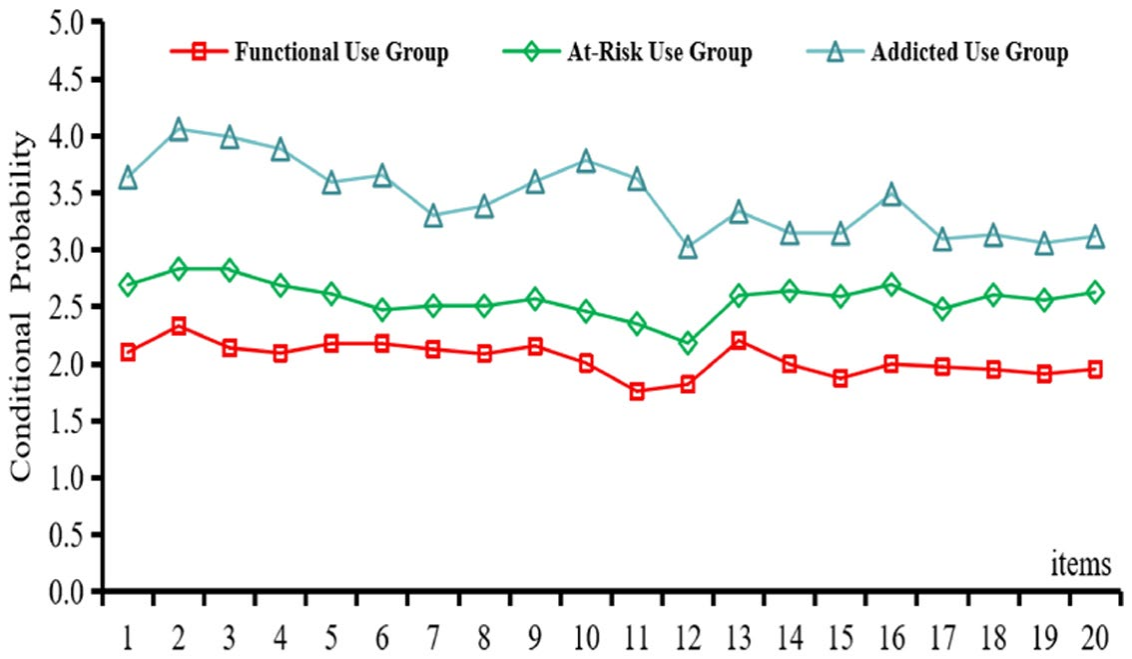
Profiles of adolescent Internet addiction.

Figure 4 illustrates the distribution of three latent Internet-addiction classes among secondary-school students. Class 1 (C1; *n* = 310, 44.9%) comprises students in thel-Use group, characterized by uniformly low scores across all items and normative patterns of Internet use. This group had a mean IAT score of 18.3, reflecting minimal symptoms. Class 2 (C2; *n* = 178, 25.7%) comprises the at-risk group, whose intermediate item scores indicate some Internet-related distress but not yet severe addiction. Their average score was 38.7, suggestive of early-stage difficulties. Class 3 (C3; *n* = 202, 29.4%) represents the Addicted-Use group, exhibiting the highest scores on every item and pronounced symptoms of Internet addiction. With a mean IAT score of 63.2 and item-level responses often exceeding 3.5, this group showed clear signs of compulsive use. These findings further corroborate substantial latent heterogeneity in Internet addiction within the adolescent population.

### 4.6 Latent type identification and path differences

**Figure 5:**
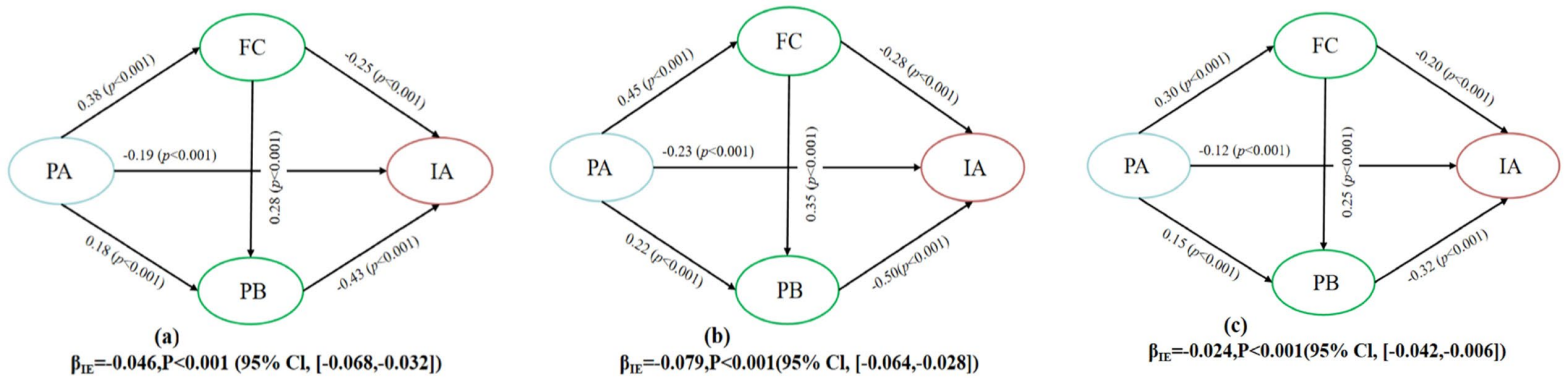
Latent profile analysis and path differences across Internet use categories. **(a)** Addicted use group β_IE_ = 0.046, *P* < 0.001 (95% CI, [−0.068, 0.032]). **(b)** At-Risk use group β_IE_ = 0.079, *P* < 0.001 (95% CI, [−0.064, 0.028]). **(c)** Functional use group β_IE_ = 0.079, *P* < 0.001 (95% CI, [−0.042, 0.006]).

This study employed latent profile analysis (LPA) to examine differences in Internet addiction and the pathways leading to it, categorizing participants into three groups: Functional Use, At-Risk Use, and Addicted Use. The results of the LPA indicated a latent categorical structure in Internet use behavior, thereby supporting hypothesis H5. We then estimated a multi-group structural equation model (SEM) using the three categories as grouping variables, as shown in Figure 5.

**Figure 6:**
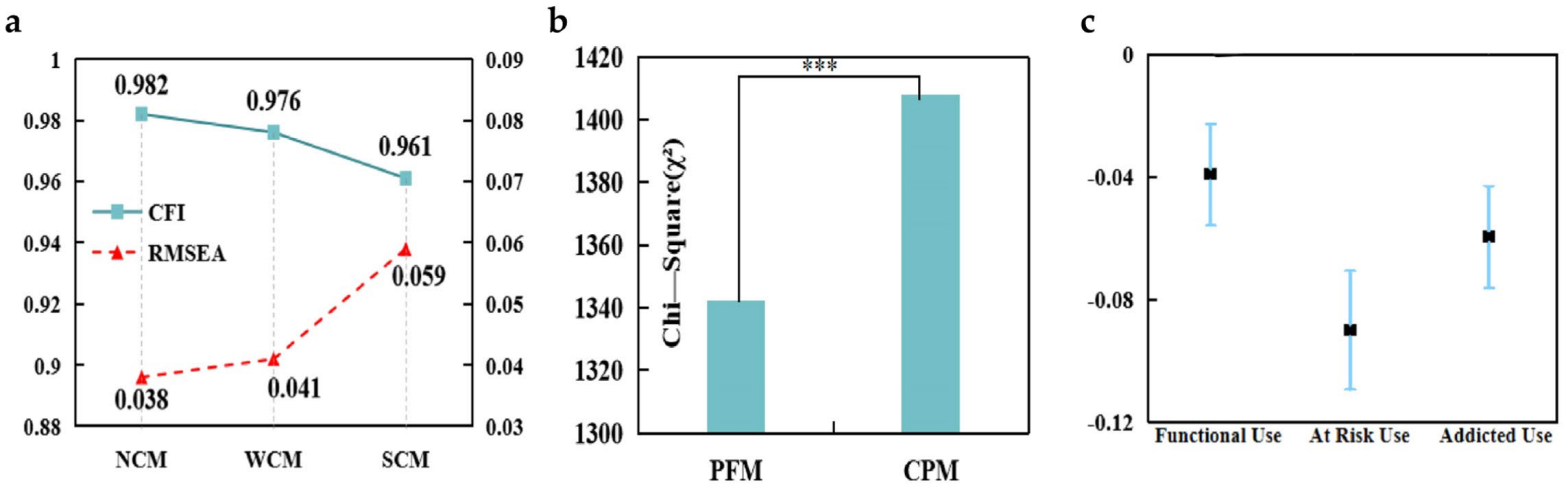
**(a)** Measurement invariance test for chained mediation pathways across groups. **(b)** Structural path differences between Internet use types. **(c)** Bootstrap estimates of chained mediation effects across Internet use groups.

To evaluate structural differences in the chained mediation pathways across groups, we first conducted a measurement-invariance test (Figure 6a). The non-constrained (NCM; CFI = 0.982, RMSEA = 0.038), weakly constrained (WCM; CFI = 0.976, RMSEA = 0.041), and strongly constrained (SCM; CFI = 0.961, RMSEA = 0.059) models all met recommended thresholds (ΔCFI < 0.01, ΔRMSEA < 0.015), thereby satisfying the prerequisites for multi-group path comparison. Figure 6b, shows that the constrained model (PFM; χ^2^ = 1407.92, df = 117) fit significantly worse than the unconstrained model (CPM; χ^2^ = 1342.18, df = 108), Δχ^2^ = 65.74, Δdf = 9, *p* < 0.001. These results demonstrate significant structural differences among Internet-use types, thereby supporting hypothesis H6.

Bootstrap estimates of chained mediation effects (Figure 6c) were −0.024 (95% CI: −0.042, −0.006) for the Functional-Use group, −0.079 (95% CI: −0.064, −0.028) for the At-Risk-Use group, and −0.046 (95% CI, −0.068, −0.032) for the Addicted-Use group. The pathway was significant in all three groups, although effect magnitudes varied, with the At-Risk-Use group exhibiting the strongest indirect effect. These findings imply that interventions should prioritize high-risk adolescents and tailor strategies to the group-specific mediation mechanisms.

## 5 Discussion and analysis

This study explored the impact of physical activity on adolescent Internet addiction, highlighting both direct and indirect effects through family cohesion and prosocial behavior. By integrating structural equation modeling with latent profile analysis, we showed that regular physical activity serves as a protective factor, reducing addictive tendencies both on its own and via strengthened family bonds and enhanced prosocial tendencies. Moreover, the relative influence of these indirect pathways differed across the three empirically derived user profiles, underscoring the importance of tailoring intervention strategies to distinct patterns of Internet use.

### 5.1 Direct moderating role of physical activity: the dual pathway mechanism of cognition and emotion

Our results indicate that higher levels of physical activity are linked to lower degrees of Internet addiction among adolescents, supporting Hypothesis H1, which aligns with prior findings (Villanueva-Blasco et al., 2021; Ma et al., 2022). Meta-analytic reviews have suggested that regular moderate-to-vigorous physical activity predicts lower digital-addiction scores in youth (Lu et al., 2025), and randomized controlled trials report sustained reductions in compulsive online behavior following structured physical activity programs. By calculating both the overall effect (β = −0.416) and the direct effect (β = −0.227), our findings add to this research, showing that physical activity has a strong protective effect on different types of internet addiction in teenagers.

Physical activity likely exerts its protective effect through several mechanisms. First, it enhances mental control: aerobic training can thicken the prefrontal cortex and improve its connectivity, which in turn strengthens decision-making and self-control—traits often compromised in individuals with Internet addiction (Srinivas et al., 2021; Bigliassi et al., 2025). Second, physical activity acts as an emotional-reward substitute by increasing the release of endorphins and dopamine, providing immediate gratification that diminishes the allure of virtual rewards (Hansen, 2017; Zhou et al., 2023). Third, PA functions as a stress buffer, raising distress tolerance and disrupting maladaptive coping cycles that drive online escapism (Carl, 2022). Finally, structured group activities foster social connectedness, satisfying relatedness needs offline and diminishing the urge for social compensation via the Internet (Chang et al., 2022).

Even though some studies, especially those based on self-reported step counts or different cultural views, have shown no results, most evidence from various types of research highlights that physical activity is an effective and easy way to prevent Internet addiction in teenagers (Alshakhsi et al., 2025).

### 5.2 The mediating role of family cohesion: emotional support and attachment regulation

Results showed that family cohesion mediated the link between physical activity and Internet addiction, supporting Hypothesis H2.

Family cohesion (FC) consists of three core dimensions: emotional support, open communication, and shared activities (Xin-cheng et al., 2023). Emotional support refers to the extent to which family members feel accepted and cared for, which helps buffer stress and promote psychological well-being (Chronister et al., 2021; Sánchez Amate and Luque de la Rosa, 2024). Open communication allows adolescents to express their concerns, seek guidance, and resolve conflicts in person, thereby decreasing their dependence on virtual forums for social and emotional needs (Procentese et al., 2019; Kapetanovic and Skoog, 2021). Shared activities—especially joint physical activity—offer structured opportunities for cooperation, mutual encouragement, and positive interaction, thereby reinforcing trust and fostering adaptive problem-solving (Ishimaru, 2019). Previous research indicates that higher levels of FC mediate the impact of parental engagement on youth behavioral adjustment (Hughes et al., 2020; Li et al., 2024a).

On the other hand, robust family cohesion directly mitigates the risk of Internet addiction by satisfying adolescents’ need for relatedness and strengthening their self-regulatory capacities (Venkatesh et al., 2019; Chen et al., 2020). Adolescents from cohesive families report lower loneliness, improved impulse control, and fewer compulsive online hours (Naeim and Rezaeisharif, 2021). Conversely, low FC is linked to emotional neglect and fragmented communication, leading youth to seek immediate reassurance and belonging in digital environments—a pattern shown to exacerbate addictive Internet use (Bickham, 2021a).

In summary, our findings demonstrate that physical activity-enhanced family cohesion constitutes a viable socio-emotional pathway through which physical activity curbs Internet addiction. By fostering emotional security and strengthening family bonds, joint physical activity creates a protective context that diminishes adolescents’ dependence on virtual gratifications.

### 5.3 The mediating role of prosocial behavior: social belonging and behavioral substitution mechanisms

Prosocial behavior significantly mediated the relationship between physical activity and Internet addiction, supporting Hypothesis H3.

This result suggests that adolescents who engage more in prosocial actions—such as helping peers, volunteering, and cooperative play—are less prone to develop addictive online habits. This aligns with the social-compensation model, which holds that individuals seek offline social support when their relatedness needs are satisfied (Kim, 2025). By fostering empathy and group identity, prosocial behavior provides real-world reinforcement and emotional fulfillment, reducing reliance on virtual social feedback (Guo et al., 2018; Lauri and Calleja, 2019).

The negative association between prosocial behavior and Internet addiction is further elaborated by its impact on emotional regulation. Helping others elevates mood through oxytocinergic and dopaminergic pathways (Wu and Hong, 2022), thereby decreasing stress and loneliness—two key drivers of compulsive online use (Li et al., 2024b). Conversely, adolescents low in prosocial tendencies may experience unmet social needs and heightened negative affect, prompting them to seek quick relief via online interaction, which can reinforce addictive patterns.

This finding expands the application of the social-substitution model, which has been used to explain substance use and self-harm in the context of digital interactions (Carrier, 2018). It suggests that Internet addiction may serve as a maladaptive strategy to compensate for insufficient real-world social engagement, highlighting prosocial behavior as a crucial protective factor.

### 5.4 Chain mediation mechanism: collaborative operation of the emotional-behavioral pathway

This study also finds that family cohesion (FC) and prosocial behavior (PB) act as sequential mediators between physical activity and Internet addiction, thereby supporting Hypothesis H4. Specifically, PA first bolsters FC—through shared routines, mutual encouragement, and open emotional exchanges—which in turn cultivates PB by reinforcing empathy, social responsibility, and group identity (Abdul Kadir, 2024; Proulx et al., 2024). Adolescents embedded in cohesive families experience heightened relatedness and trust, making them more inclined to engage in helping behaviors that satisfy their need for social affiliation offline. These helpful actions serve as a real-life replacement for online rewards: by offering real-world benefits like feeling good and gaining friends’ approval, PB weakens the desire for online interactions and lessens excessive Internet use (Giordano, 2021). In our study, this connected process had an indirect effect of −0.042, showing that the combined social and emotional flow—PA → FC → PB—while smaller than individual factors, is still important. In simple terms, this sequence aligns with Self-Determination Theory, which suggests that meeting the need for connection with family helps young people adopt positive social behaviors, leading to better control over their Internet use (House, 2018; Barton et al., 2020). In contrast, the absence of FC or PB leaves adolescents vulnerable to virtual surrogates for connection, perpetuating addictive patterns (Begun and Murray, 2020). These findings suggest that physical activity is associated with lower scores of Internet addiction, highlighting the importance of integrating family and peer systems when promoting physical activity as a protective factor.

### 5.5 Latent profile analysis of internet-use profiles and its moderating effects

Using LPA, we identified three distinct Internet-use profiles—Functional Use (31.2%), At-Risk Use (49.5%), and Addicted Use (19.3%)—thereby supporting Hypothesis H5 and aligning with similar typology findings reported in previous cross-cultural research. For example, Kwak et al. (2022) identified similar proportions (28–52–20%) of Internet use severity among Korean adolescents, while Martins and Rodrigues reported corresponding distributions (33–47–20%) in a sample from Portugal (Martins et al., 2022). These studies suggest that adolescents tend to form similar patterns of Internet use regardless of cultural background, reflecting a broader global trend. In this group, adolescents who used the Internet functionally had the lowest scores for Internet Addiction (IA) (M = 1.45, SD = 0.32), showing they used the Internet in a healthy way, similar to the low-risk group identified by Krisztian Kapus in Hungarian youth (Kapus et al., 2021), who had scores below the levels for IA diagnosis. In contrast, adolescents at risk showed a moderate level of problematic Internet use (*M* = 3.27, SD = 0.48), which matches the “subclinical” risk profiles found by Aziz and Chemnad in their study of adolescents from Qatar (Aziz et al., 2024). Finally, the Addicted Use group, representing the highest IA severity (*M* = 5.12, SD = 0.56), mirrors the clinical high-risk profiles documented in studies of adolescents diagnosed with Internet addiction (Gansner et al., 2019). These findings support the trustworthiness of the three-profile solution in various groups of teenagers and highlight that Internet addiction patterns are similar across different cultures.

The number of participants in the three groups was not equal, with at-risk use constituting the biggest part of the sample (49.5%), then functional use (31.2%), and addicted use (19.3%). This uneven distribution matches earlier research, like the study by Park and Lee (2022), which showed that the middle-risk group—similar to our At-Risk Use category—often had the largest number of adolescents. This pattern suggests that a majority of adolescents may fall into the moderate-risk category, marked by some problematic behaviors without reaching clinical levels of addiction.

Several factors may explain this profile distribution. Digital literacy and socioeconomic factors significantly shape adolescents’ engagement with digital technologies. As noted by Sechi, adolescents with higher digital literacy may be more adept at managing their screen time and mitigating addiction risks (Sechi et al., 2025), while those from lower socioeconomic backgrounds might have limited access to education on healthy Internet use, thereby increasing their likelihood of falling into the At Risk Use or Addicted Use profiles. Furthermore, motivational factors also play a crucial role in profile classification, as adolescents with intrinsic motivation for online activities are more likely to fall into Functional Use, while those seeking external validation or experiencing emotional distress may gravitate toward the At Risk Use or Addicted Use profiles (Link and Baumann, 2020; Pogozhina et al., 2020).

The distribution patterns outlined offer helpful details about prevention and intervention strategies for Internet addiction. The at-risk use profile, which includes nearly half of the sample, suggests that there are early-stage interventions targeting adolescents who display moderate levels of Internet use. The result suggests that efforts should focus on enhancing digital literacy, improving emotional regulation, and addressing motivational factors that contribute to excessive screen time. On the other hand, adolescents in the Functional Use group may benefit from developing healthy online habits and engaging more in positive offline activities. In contrast, those with addictive use are likely to require more intensive interventions that tackle deeper emotional issues and offer stronger support systems to alleviate the impacts of excessive online engagement.

The subsequent analysis using multi-group Structural Equation Modeling (SEM) revealed significant differences in the mediation pathways among the three profiles (Δχ^2^ = 65.74, *p* < 0.001), thereby supporting Hypothesis H6. The mediation pathway linking physical activity, family cohesion, and prosocial behavior to Internet addiction was strongest in the at-risk use profile (β = −0.079), moderate in the addicted use profile (β = −0.045), and weakest in the functional use profile (β = −0.028). This unexpected pattern aligns with the Cognitive–Motivation Hierarchy Model, indicating that individuals with moderate self-regulation abilities, like those in the at-risk category, are more susceptible to the influence of social and emotional support compared to those in high-risk or low-risk categories (Lee and Jeong, 2024). In contrast, adolescents with significant Internet dependency may find it challenging to benefit from these interventions unless they first address their emotional regulation and family dynamics (Fan, 2022). The input text is clear and maintains a consistent tone. However, it can be slightly refined for improved readability and coherence. Here’s a revised version: Functional use Adolescents show minimal benefit from these interventions, as they already demonstrate healthy Internet use patterns. These findings underscore the importance of targeted, profile-specific interventions. At-risk-use adolescents require a combination of family cohesion and prosocial behavior programs, while addicted-use adolescents may need more intensive emotional support and regulation strategies. For functional use by adolescents, the focus should be on maintaining their healthy behaviors.

These results underscore the importance of matching intervention strategies to adolescents’ specific usage profiles. For those in the At-Risk group, strengthening family cohesion and fostering prosocial behaviors can help prevent escalation into problematic use. Adolescents already exhibiting addictive patterns will benefit most from programs that target emotional regulation, reinforce healthy family relationships, and build robust offline support networks. By contrast, youths in the Functional-Use group require only light-touch interventions that bolster their existing positive habits both online and off. Tailoring our approach in this way promises a more precise and effective response to the diverse needs of adolescents at different stages of Internet engagement.

## 6 Conclusion

The present study, focusing on adolescent internet addiction, has explored the factor structures of physical activity, family cohesion, and prosocial behavior, revealing their predictive effects. In addition, this research applies Latent Profile Analysis (LPA) to identify distinct internet-use profiles among adolescents and examines their moderating effects. Based on these findings, the study also highlights potential directions for future research. Regarding the factor structures, the study identified that physical activity, family cohesion, and prosocial behavior each exhibit unique characteristics. Physical activity was found to directly inhibit internet addiction, while family cohesion and prosocial behavior were best represented as mediating factors in this relationship. Specifically, physical activity had a significant direct effect on internet addiction (β = −0.416, p < 0.001), with indirect effects through family cohesion (−0.065, 15.7% of total effect) and prosocial behavior (−0.083, 19.9% of total effect). Furthermore, a chained pathway from family cohesion to prosocial behavior (−0.042, 10.1% of total effect) was observed, suggesting a complex interplay between these factors. Latent Profile Analysis (LPA) revealed three distinct profiles of adolescent internet use: Functional Use, At-Risk Use, and Addicted Use. Multi-group Structural Equation Modeling (SEM) indicated that the magnitude of the indirect effects varied across these profiles. The At-Risk Use group exhibited the strongest mediation, emphasizing the importance of targeted interventions for this subgroup. These findings highlight the significant role of physical activity in reducing adolescent internet addiction through socio-psychological mechanisms. The study also underscores the value of identifying subgroups using LPA, which provides a more nuanced understanding of adolescent internet behaviors. Tailored interventions can be developed based on these profiles, with a focus on strengthening family cohesion and promoting prosocial behavior in at-risk adolescents. Additionally, the study suggests potential avenues for future research, such as the incorporation of multimodal data analysis and the exploration of other mediating variables, including motivation and digital literacy. The use of longitudinal or experimental designs would further validate the causal relationships observed in this study.

These findings underscore the value of identifying subgroups using LPA, which provides a more nuanced understanding of adolescent internet behaviors. Tailoring intervention intensity and content based on user typology is essential, as adolescents with different profiles may exhibit varying levels of vulnerability and thus require differentiated psychological support strategies. Recognizing these distinctions can enhance the precision of prevention programs and increase their effectiveness in real-world applications.

In addition, practical applications should be considered when translating research into school-based interventions. Schools could implement physical education curricula that emphasize cooperative activity, thereby fostering both peer connectedness and family participation. By enhancing real-life social engagement through structured group interaction, such programs may help mitigate problematic internet use while promoting prosocial development.

## 7 Study limitations and suggestions for future research

Despite the significant contributions of this study, several limitations must be acknowledged to appropriately interpret the findings and inform future research directions.

First, the cross-sectional nature of the research restricts the ability to establish causal relationships among variables. While the findings suggest meaningful associations, future research employing longitudinal or experimental designs is necessary to better ascertain causal inferences and temporal dynamics among physical activity, socio-psychological factors, and Internet addiction. Additionally, although family cohesion and prosocial behavior accounted for significant portions of the mediation effects observed, other unmeasured or latent variables might influence these relationships. Factors such as emotional resilience, self-esteem, and digital literacy, which could further explain adolescents’ susceptibility to Internet addiction, were not assessed. Future research should incorporate these potential mediators to achieve a more comprehensive understanding.

Second, the generalizability of the findings is limited by the sample composition. The data were collected from three public middle schools in Shandong Province, characterized by geographic and institutional homogeneity. Consequently, the findings may not generalize adequately to adolescents from other regions or to those attending private or vocational schools. To enhance external validity, subsequent research should aim to include more diverse samples across various geographic locations and educational contexts.

Third, reliance on self-report questionnaires introduces the possibility of social desirability bias. Participants might have overreported socially desirable behaviors such as physical activity and prosocial tendencies, or conversely, underreported problematic behaviors like excessive Internet use. Such biases may distort observed relationships, thus warranting caution in interpreting these findings. Future studies could utilize multi-informant methods or objective measures, such as accelerometers for physical activity or software monitoring for Internet usage, to mitigate these biases and provide more accurate assessments.

Fourth, this study did not differentiate between various types and content of screen time. Active engagement with screens (e.g., educational or interactive use) is typically goal-directed and cognitively stimulating, while passive screen time (e.g., video watching) tends to promote disengagement and habitual use. Prior research indicates distinct psychological outcomes associated with these two types of engagement, with active use generally resulting in fewer negative effects (Veraksa et al., 2021). Moreover, the specific content, such as online gaming versus social media usage, likely triggers unique motivational and emotional responses. Online gaming, for instance, has been strongly associated with compulsive behavior and reward-seeking patterns, whereas social media usage predominantly relates to social comparison and identity management concerns (Maheux et al., 2025). Future research should therefore disaggregate screen usage both by mode (active vs. passive) and content domain (gaming vs. social media), enabling more precise identification of distinct pathways leading to Internet addiction and informing targeted interventions.

Fifth, the findings’ applicability might be influenced significantly by cultural contexts. This study was conducted within an East Asian, specifically Chinese, sociocultural framework characterized by an emphasis on interdependence, obedience, and collective identity within families. Such cultural norms might amplify the psychological and behavioral influences of family cohesion, potentially limiting the generalizability of the mediation model to Western or more individualistic cultures. Cross-cultural comparative studies are necessary to test the robustness and applicability of the proposed theoretical mechanisms across diverse sociocultural environments.

In addition to the mediators already discussed, future research might consider examining other psychological and behavioral factors that could further clarify the complex pathways linking physical activity to adolescent Internet addiction. For instance, variables such as parental monitoring, peer influences, or school engagement may significantly impact adolescents’ online behaviors. Integrating these additional dimensions could enhance the theoretical breadth and practical effectiveness of intervention strategies.

In summary, despite its limitations, this study contributes substantially to understanding the psychological mechanisms underlying adolescent Internet addiction. By identifying distinct user profiles and clarifying multi-level mediating mechanisms, the findings offer critical insights for developing tailored intervention strategies. Future research addressing these identified limitations will further enhance theoretical clarity, empirical robustness, and practical applicability in combating Internet addiction among adolescents.

## Data Availability

The raw data supporting the conclusions of this article will be made available by the authors, without undue reservation.
