# Correction: The relationship between physical activity and Internet addiction among Chinese adolescents: exploring latent profile analysis and multi-level mediating mechanisms

**DOI:** 10.3389/fpsyg.2025.1680220

**Published:** 2025-08-21

**Authors:** Jiamin Zhu, Yutong Zhai, Xiaotong Yuan, Zhiyong Zhang, Xiaoping Meng

**Affiliations:** ^1^Graduate School, Shandong Sport University, Jinan, China; ^2^College of Economics and Management, Nanjing University of Aeronautics and Astronautics, Nanjing, China; ^3^Institute of Physical Education and Social Sciences, Shandong Sports University, Jinan, China; ^4^School of Sport Management, Shandong Sport University, Jinan, China

**Keywords:** Internet addiction, physical activity, family cohesion, prosocial behavior, latent profile analysis

The order of authors in the author list of the published paper was erroneously given as:

Jiamin Zhu^1†^, Yutong Zhai^2†^, Xiaoping Meng^3^, Zhiyong Zhang^3^, Xiaotong Yuan^1*^

The correct author list reads:

Jiamin Zhu^1†^, Yutong Zhai^2†^, Xiaotong Yuan^1^, Zhiyong Zhang^3^ and Xiaoping Meng^3, 4*^

Affiliation School of Sport Management, Shandong Sport University, Jinan, China was omitted from the paper. This affiliation has now been added for author Xiaoping Meng.

Author Xiaotong Yuan was erroneously assigned as corresponding author. The correct corresponding author is Xiaoping Meng.

The original version of this article has been updated.

